# Effects of GLP-1 and GIP on cholinergic-induced contractility in isolated jejunal muscle from obese patients with and without type 2 diabetes mellitus

**DOI:** 10.3389/fphys.2025.1734360

**Published:** 2026-01-05

**Authors:** Mantas Malinauskas, Darius Stukas, Kristina Rysevaite-Kyguoliene, Rita Gudaityte, Limas Kupčinskas, Anna Casselbrant, Lina Jankauskaite, Almantas Maleckas

**Affiliations:** 1 Institute of Physiology and Pharmacology, Lithuanian University of Health Sciences, Kaunas, Lithuania; 2 Institute for Digestive Research, Lithuanian University of Health Sciences, Kaunas, Lithuania; 3 Institute of Anatomy, Lithuanian University of Health Sciences, Kaunas, Lithuania; 4 Department of Surgery, Lithuanian University of Health Sciences, Kaunas, Lithuania; 5 Department of Surgery, Institute of Clinical Sciences, Sahlgrenska Academy, University of Gothenburg, Gothenburg, Sweden

**Keywords:** DPP-4 activity, gastric inhibitory polypeptide, gastrointestinal motility, glucagon like peptide-1, type 2 diabetes mellitus

## Abstract

**Background:**

Intestinal dysmotility in type 2 diabetes mellitus (T2DM) may involve impaired cholinergic and incretin-mediated regulation. This study compared cholinergic-induced jejunal contractility and evaluated the effects of Glucagon like peptide-1 (GLP-1) and Gastric inhibitory polypeptide (GIP) in relation to the expression of these peptides, their receptors, and Dipeptidyl peptidase 4 (DPP-4) in jejunal muscle of obese patients with and without T2DM.

**Methods:**

Jejunal samples were collected from 32 obese patients undergoing bariatric surgery (14 with and 18 without T2DM). Jejunal muscular tissue was examined for expression of GLP-1, GIP, and for expression and localization of DPP-4 and incretin receptors (GLP-1R and GIPR). In addition, DPP-4 enzymatic activity was quantitatively assessed. Contractility of circular and longitudinal muscle strips was assessed *in vitro* following bethanechol stimulation, with or without GLP-1 or GIP.

**Results:**

GLP-1 receptors were detected in smooth muscle nuclei and enteric ganglia, while GIP receptors localized to both muscle layers. DPP-4 was present in neural and muscular compartments. In T2DM, GIPR and DPP-4 expression and activity were increased, while GIP protein was reduced. GLP-1 protein levels tended to be higher. Longitudinal muscle contractility independent of neural input was reduced in T2DM. GLP-1 selectively inhibited circular muscle contractions in both groups, whereas GIP had no effect.

**Conclusion:**

This study demonstrates that reduced cholinergic activity in longitudinal muscle, lower GIP, and increased GLP-1 in T2DM indicate a shifted local incretin environment that may collectively suppress jejunal contractility.

## Introduction

Obesity is the leading cause of type 2 diabetes mellitus (T2DM) and is responsible for the increasing prevalence of T2DM worldwide. Genetic and epigenetic factors together with visceral fat accumulation, pancreas β cell dysfunction and changes in gut brain axis are responsible for the development of both diseases (Chandrasekaran and Weiskirchen, 2024). Due to progressive nature, T2DM affects different human organs and tissues leading to major complications such as myocardial infarction, stroke, peripheral arterial ischemia or end stage renal disease. Gastrointestinal symptoms are common among individuals with T2DM. Autonomic neuropathy together with changes in microbiota, enteric nervous system, hormonal homeostasis and smooth muscle myopathy are responsible for T2DM gastrointestinal dysfunction (Portincasa et al., 2022). Recent evidence suggests that T2DM and irritable bowel syndrome (IBS) have bidirectional association, with increased risk of IBS among patients with T2DM and higher risk of T2DM among patients with IBS (Zhou et al., 2024).

Acetylcholine is a primary excitatory neurotransmitter, which regulates gastrointestinal motility through M1-5 muscarinic receptors either directly on smooth muscles cells or indirectly through the enteric nervous system. M2 outnumbers M3 in gastrointestinal smooth muscle cells, however *in vitro* studies suggest that M3 receptors are primarily responsible for mediating excitatory effects of acetylcholine on gastrointestinal contractility (Bharucha et al., 2010). In patients with IBS, muscarinic antagonists are effective in treating gastrointestinal symptoms such as abdominal cramps (Ruepert et al., 2011). Moreover, in humans M3 receptors regulate small intestinal and colonic transit (Bharucha et al., 2010). Studies on diabetic rats have shown increased function of neural M2 receptors in their gut, which reduces acetylcholine release from parasympathetic nerves and may contribute to decreased gut motility (Coulson et al., 2002). However, changes in these receptors in humans with diabetes remain underexplored.

On the other hand, gut peptides, such as incretins, are important regulators of gastrointestinal motility. Glucagon-like peptide 1 (GLP1) is secreted by intestinal L-cells, regulates glucose homeostasis and has inhibitory effect on gastrointestinal motility (Amato et al., 2010). It decreases gastric emptying and intestinal motility and contributes to the “ileal brake” mechanism (Hellström et al., 2008; Nauck and Meier, 2018). Another incretin, glucose-dependent insulinotropic peptide (GIP), is produced by K cells of duodenal and jejunal mucosa (Edholm et al., 2009). In addition to its insulinotropic activity GIP has potential to inhibit small bowel motility in rodents (Nauck and Müller, 2023). In T2DM insulinotropic potency of GIP and GLP-1 is reduced (Halim et al., 2018), but the impact on motility is preserved (Nauck and Müller, 2023). Moreover, inhibiting motility incretins may mitigate some IBS symptoms such as abdominal cramps. Few studies so far have explored the impact of incretins on small bowel motility in humans with obesity (Halim et al., 2018), but the studies on patients with T2DM are lacking. Importantly, both GLP-1 and GIP are rapidly inactivated by the enzyme dipeptidyl peptidase-4 (DPP-4), which may modulate their biological activity not only systemically but also locally in the gastrointestinal tract (Deacon, 2020; D’Alessio, 2011). Differences in DPP-4 activity and incretin degradation between obese individuals with and without T2DM may therefore contribute to distinct effects on intestinal motility, which remain insufficiently explored.

This study aimed to compare jejunal contractile responses to muscarinic receptor stimulation between obese patients with type 2 diabetes (OB+/DM+) and obese patients without diabetes (OB+/DM−), and to assess the effects of GLP-1 and GIP on jejunal contraction in relation to jejunal muscle expression of these peptides, their receptors, and DPP-4.

## Materials and methods

### Study participants

This study included patients diagnosed with morbid obesity who had undergone laparoscopic Roux-en-Y gastric bypass surgery. Participants were eligible for inclusion if they were aged between 18 and 65 years and had either a BMI ≥40, or a BMI ≥35 accompanied by obesity-related comorbidities. Exclusion criteria comprised treatment with DPP-IV inhibitors and/or GLP-1 receptor agonists, a diagnosis of type 1 diabetes or any non–type 2 form of diabetes, active severe psychiatric disorders, alcohol or substance abuse, and end-stage organ failure. Patient health status was assessed through a structured interview. The diagnosis of type 2 diabetes mellitus (T2DM) was established based on either a fasting plasma glucose level ≥7.0 mmol/L, HbA1c ≥6.5%, or the current use of oral antidiabetic agents or insulin therapy. Hypertension was defined as systolic/diastolic blood pressure ≥140/90 mmHg or ongoing treatment with antihypertensive agents. Dyslipidemia was diagnosed if total cholesterol exceeded 5.2 mmol/L (200 mg/dL), triglycerides were >2.26 mmol/L (200 mg/dL), or HDL cholesterol was <1.04 mmol/L (40 mg/dL) in men and <1.3 mmol/L (50 mg/dL) in women, or if patients were receiving statin therapy. The study included 32 participants (7 men and 25 women) with a mean age of 45.6 ± 13.6 years and a mean BMI of 46.2 ± 7.7 kg/m^2^. The cohort was stratified into two groups: patients with diabetes (OB+/DM+) and those without diabetes (OB+/DM−). Patients in OB+/DM+ group who were on metformin treatment discontinued metformin at least 3 days prior to the surgery.

### Preparation of surgically resected intestinal tissue

Full-thickness jejunal specimens were obtained from patients undergoing Roux-en-Y gastric bypass surgery for treatment of morbid obesity. Tissue samples were resected between the gastroentero-entero anastomosis and the entero-entero anastomosis as the bowel loop was divided to create the Roux-en-Y construction. Following resection, the mucosal and submucosal layers were carefully separated from the underlying muscularis propria using sharp dissection techniques. A portion of the isolated muscular layer was immediately snap-frozen in liquid nitrogen and subsequently stored at −80 °C for protein analysis. The remaining tissue was either fixed or placed in Krebs buffer for use in *in vitro* contractility experiments.

### Immunofluorescence

Tisue samples obtained from patients were washed in 0.1 M phosphate-buffered saline (PBS) and then fixed with 4% paraformaldehyde solution (PFA) in 0.1 M PBS (pH = 7.4) for 2–3 h, depending on the sample size. After, again washed in PBS, then immersed in 30% sucrose in 0.1M PBS containing 0.1% sodium azide in 4 °C for 24 h. Following cryoprotection, jejunal muscular tissue was frozen at −30 °C using a tissue-freezing medium (Triangle Biomedical Sciences, United States) for sectioning. Next, tissues were sectioned into 18 µm slices using a cryomicrotome HM 560 (Microm, Germany) at −22 °C, mounted onto Superfrost Plus microscope slides (Menzel Glaser, Germany), and dried at room temperature for 30 min. For immunohistochemical permeabilization, sections were incubated for 40 min In a solution containing 9% dimethyl sulfoxide (DMSO) and 1% Triton X-100 in 0.1M PBS. After 3 × 10 min washing in PBS, sections were applied with 5% normal donkeys serums (NDS) (Jackson ImmunoResearch Laboratories, West Grove, PA, United States), immunohistochemical procedures were continuing applying primary antibodies: rabbit polyclonal anti-GIPR (Cusabio, United States, CSB-PA009438ESR1HU; 1:500); rabbit polyclonal anti-GLP1R (Cusabio, United States, CSB-PA183226; 1:500); rabbit anti-DPP4 (CBS-PA06229A0Rb, Cusabio, United States; 1:500), mouse monoclonal anti-PGP 9.5 (Abcam, United Kingdom, ab8189; 1:500); mouse monoclonal anti-alpha smooth muscle actin (Abcam, United Kingdom, ab7817; 1:500), overnight at 4 °C. After washing in PBS, secondary antisera: donkey anti-rabbit (Millipore, Burlington, MA, AP182C, conjugated with Cy3 fluorochrome), and donkey anti-mouse (Millipore, Burlington, MA, AP192F, conjugated with FITC fluorochrome) were applied on the sections. Finally, specimens were washed three times for 5–8 min in 0.1 M PBS mounted with a Vectashield Mounting Medium (Vector Laboratories, Inc., Burlingame, CA, United States), cover slipped and sealed with clear nail polish. Primary antibody (negative) controls were processed in parallel to inspect signal specificity. Preparations were imaged using confocal laser scanning microscope LSM 700 with the software package ZEN 2010B SP1 (Carl Zeiss, Jena, Germany).

### Protein analysis

#### Lysate preparation

Jejunal muscle samples stored at −80 °C were thawed and weighed. Tissues were rinsed with chilled phosphate-buffered saline (PBS), finely minced on ice, and mechanically disrupted using Lysing Matrix D tubes (MP Biomedicals) in the presence of ice-cold RIPA buffer (Thermo Scientific). Homogenization was carried out on the MagNA Lyser system (Roche, Germany) by applying three brief cycles at 6,000 rpm, each separated by a 15-s cooling interval. Following homogenization, lysates were incubated on ice for 30 min with intermittent vortexing. The homogenates were then centrifuged at high speed to remove debris, and the clarified supernatants were aliquoted and stored at −80 °C for subsequent analysis.

#### Quantification of protein levels

The concentrations of GLP-1 and GIP in tissue supernatants were assessed using commercially available ELISA kits (GLP-1: E-EL-H6025; GIP: E-EL-H2061; both from Elabscience, China). All procedures, including plate preparation and reagent handling, were carried out in accordance with the manufacturer’s protocols. Optical density at 450 nm was recorded using a Multiskan GO microplate reader (Thermo Fisher Scientific, software version 1.00.40).

#### Western blot analysis

The tissue samples of OB+/DM− and OB+/DM− patients were homogenized and lysed using a Bead Mill MagNA Lyser (Roche, Basel, Switzerland), Lysing Matrix D (MPbiomedicals, Santa Ana, CA, MP116913500) tubes and RIPA lysis buffer (abcam, Cambridge, United Kingdom, ab156034) supplemented with protease inhibitors (Thermo Scientific, Waltham, MA, United States, 78441). The acquired lysates were then centrifuged at 10,000 × G for 10 min at 4 °C after which the supernatants were used for the protein analysis. The protein concentration was measured using BCA protein assay kit (Thermo Scientific, Waltham, MA, United States, 23227) according to manufacturer’s protocols. The protein samples were mixed with sample buffer (NuPage LDS Sample buffer (Invitrogen, Carlsbad, CA, United States; NP0007)) and reducing agent (NuPage sample reducing agent (Invitrogen, Carlsbad, CA, United States; NP0009)), heated at 97 °C for 5 min before loading onto the gel. 30 μg samples were subjected to 4%–12% sodium dodecyl sulfate–polyacrylamide gel electrophoresis (SDS–PAGE) and subsequently transferred to polyvinylidene fluoride (PVDF) membranes (Merck Millipore, MA, United States, IPVH00010). After transferring, the membrane was blocked using WesternBreeze Chemiluminescent Kit (Invitrogen, Carlsbad, CA, United States; WB7104). The membrane was then subjected to primary antibodies overnight at 4 °C. The following primary antibodies were used: mouse monoclonal anti-GAPDH (Invitrogen, Carlsbad, CA, United States; AM4300; 1:3000); rabbit polyclonal anti-GIPR (Cusabio, United States, CSB-PA009438ESR1HU; 1:1000); rabbit polyclonal anti-GLP1R (Cusabio, United States, CSB-PA183226; 1:1000); rabbit polyclonal anti-DPP4 (Cusabio, United States, CBS-PA06229A0Rb). Subsequent washing and secondary antibody incubation and visualization were conducted using WesternBreeze Chemiluminescent Kit. The membrane was documented using Bio-Rad documentation system (Bio-Rad, Hercules, CA, United States).

### DPP-4 enzyme assay

DPP4 activity was determined in jejunal tissue supernatants using the DPP4 activity assay kit (Sigma-Aldrich, United States, MAK088). Tissue samples (10 mg) were homogenized in 4 volumes of ice-cold DPP4 assay buffer and centrifuged at 13,000 × g for 10 min. The supernatants (50 µL) were used for analysis. For the assay, 10 µL of assay buffer was added to each well, while 10 µL of inhibitor was added to blank wells, followed by incubation at 37 °C for 10 min. The reaction was initiated by adding 40 µL of a substrate-containing master mix (38 µL assay buffer and 2 µL H-Gly-Pro-AMC substrate). After incubation for 5 min at 37 °C, fluorescence was measured using a spectrofluorometer (Thermo Scientific Fluoroskan Ascent Microplate Fluorometer). A standard curve for AMC was generated using 0–100 pmol/well AMC in assay buffer. DPP4 activity in microunits/mL is calculated by converting pmole of AMC released per minute to µmole per minute per mL, where 1 microunit corresponds to 1 µmole of AMC hydrolyzed per minute at 37 °C.

### *In vitro* contractility assessment

After resection, tissue was placed in cold oxygenated (95% O_2_–5% CO_2_) Krebs solution (4.69 mM KCl, 2.52 mM CaCl2, 1.16 mMMgSO4, 1.01 mM NaH2PO4, 25 mM NaHCO3, 118.07 mM NaCl, 11.10 mM glucose) chilled on ice. The muscle tissue of jejunum, including the circular and longitudinal layers, were separated from the mucosa/submucosa by means of sharp dissection and then cut into approximately 2 × 10 mm strips oriented along either the longitudinal or circular muscle axis, with both layers preserved to maintain the integrity of the myenteric plexus. Jejunal muscular strips were submerged in warm (37 °C) aerated Krebs solution in individual 25 mL wells (Radnoti organ bath, AD instruments Pty, AU) anchored by a metallic hook at the lower end and attached by silk suture to a force transducer for isometric recording of muscular activity (PowerLab®, AD Instruments Pty). The muscular strips were initially stretched to 1 g and left to equilibrate for 45 min before chemical stimulation, replacing the Krebs solution every 15 min to establish spontaneous contractions at a stable frequency.

Bethanechol-induced (BTCH) contraction of jejunal muscle strips was assessed by constructing a concentration–response curve using cumulative additions of BTCH (10^−8^–10^−3^ M) (Sigma–Aldrich, United States, CAS 590-63-6) to determine the EC_50_ and a near-maximal effective concentration. To standardize further comparative experiments between experimental groups, a near-maximal BTCH concentration (10^−4^ M), as identified from the plateau phase of the dose–response curve, was used for testing jejunal contractility across two patients’ cohorts: OB+/DM─ and OB+/DM─ and included in the final testing protocol.

Jejunal muscle strips were pretreated with GLP-1 (7-36) amide (10^−7^ M) (Tocris, United Kingdom, 107444-51-9), GIP (1-39) (10^−7^ M) (Tocris, United Kingdom, 2257), or Krebs solution (vehicle) for 2 min, followed by administration of BTCH (10^−4^ M) with a 5-min contact period to elicit a maximal reference contraction. In three experiments, tetrodotoxin TTX (10^−7^ M) (Carl Roth, DE, 4368-28-9) was applied 15 min prior to peptide administration to assess the role of neural input. To evaluate the maximal effect of GLP-1 and GIP on BTCH-induced contraction, the highest technically feasible concentration (10^−7^ M) was used therefore, concentration–response relationships for these peptides were not established. Contractile responses were expressed as a percentage of the maximal BTCH-induced contraction. Each muscle strip was exposed to only one treatment condition, and all strips within a single experimental run were obtained from the same individual.

### Data analysis statistics

The Shapiro–Wilk test confirmed that the data sets were not normally distributed. Consequently, the Mann–Whitney U test was used to compare the means presented in Table 1, as well as DPP-4 activity and the levels of GLP-1, GIP, and their receptors between the OB+/DM+ and OB+/DM− groups. In addition, the Mann–Whitney U test was applied to assess differences in BTCH-evoked contraction between the OB+/DM+ and OB+/DM− groups, including responses measured after GLP-1, GIP, and vehicle treatment. Statistical analyses were conducted using SPSS for Windows (version 23.0; IBM Corp., Armonk, NY) and GraphPad Prism (version 9; GraphPad Software, San Diego, CA). P-value <0.05 was considered indicative of statistical significance.

**TABLE 1:** Patients’ characteristics.

Characteristics	OB+/DM+ (n = 14)	OB+/DM− (n = 18)	^a^ *P-*value
Sex F/M	4/10	3/15	0.419
Age, years^b^ (SD)	52.6 (11.2)	40.2 (13.0)	0.008**
BMI kg/m^2^ (SD)	44.9 (4.9)	47.1 (8.9)	0.417
Fasting glucose	6.88 (1.28)	5.26 (0.37)	0.014**
HbA1c	6.28 (0.97)	5.34 (0.45)	0.061
Diabetes duration	4.30 (6.0)	-	
Medications for diabetes n (%)			
Biguanide (metformin	11 (78.6)	-	
Secretagogue	1 (7.1)		
SGLT2 inhibitors	1 (7.1)		
Insulin	1 (7.1)		
Hypertension ^c^y/n	14/0	9/9	0.002**
Dyslipidemia y/n	7/7	4/14	0.101

^a^ Significant difference are denoted by (**P ≤ 0.01) (Mann-Whitney U-test).

^b^ Standard deviation (SD).

^c^ y/n (yes/no) – presence or absence of indicated condition.

## Results

Table 1 presents a comparison of clinical characteristics between patients with obesity and type 2 diabetes (OB+/DM+) and those with obesity but without diabetes (OB+/DM−). Patients in the OB+/DM+ group were significantly older (52.6 ± 11.2 vs. 40.2 ± 13.0 years, *P* = 0.008), and had higher fasting glucose levels (6.28 ± 1.28 vs. 5.34 ± 0.45 mmol/L, *P* = 0.014). Hypertension was more prevalent in the OB+/DM+ group (14/0 vs. 9/9, *P* = 0.002). No significant differences were observed in sex, BMI, HbA1c or dyslipidemia prevalence between the groups. Diabetes medications in the OB+/DM+ group included biguanides (78.6%), secretagogues, SGLT2 inhibitors and insulin (7.1% of each).

### Localization of GLP-1 and GIP receptors and DPP-4 in jejunal muscular tissue

**FIGURE 1:**
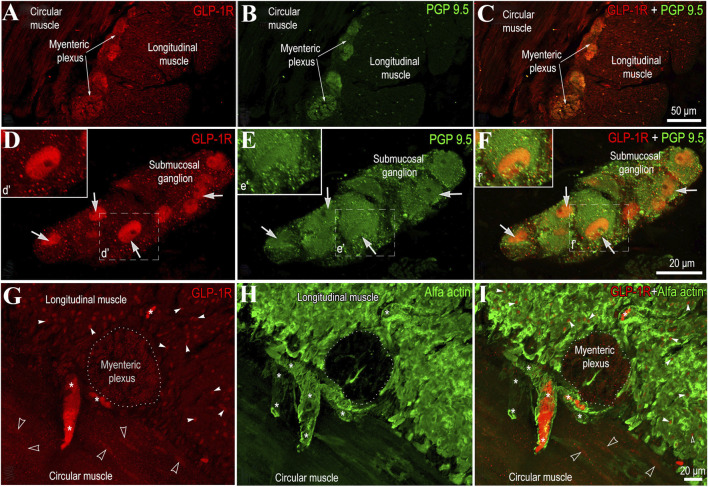
Representative images showing GLP-1 receptor localization in the human jejunal muscular tissue. **(A–C)** show transverse sections of the jejunal wall immunolabeled for GLP-1 receptor (GLP-1R, red) and the pan-neuronal marker PGP 9.5 (green), including the longitudinal and circular muscle layers and the interposed myenteric plexus. **(C)** presents a merged image of GLP-1R and PGP 9.5 labeling. **(D–F)** depict the submucosal region, including the submucosal ganglia, labeled for GLP-1R **(D)**, PGP 9.5 **(E)**, and their merged expression **(F)**. Insets (d′–f′) represent higher-magnification views of boxed areas. **(G–I)** provide a higher-magnification view of the inner and outer muscle layers (stained with an anti-smooth muscle antibody) and the myenteric plexus (outlined with a dashed area) between them. White asterisks indicate blood vessels, some of which are filled with blood cells that also show strong GLP-1R positivity. Empty and white arrowheads point to the nuclei of circular and longitudinal myocytes, respectively.

Immunofluorescence analysis showed that GLP-1 receptors (GLP-1R) are most abundant in the myenteric plexus (Figures 1A–C,G–I) and the submucosal ganglia (Figures 1D–F). Their distribution in neural tissue is uneven, with GLP-1R primarily concentrated in the nuclei of neurons (Figures 1D,F). Additionally, these receptors are present in both circular and longitudinal muscle layers (Figures 1A–C,G–I), with the highest concentration observed in the nuclei of smooth muscle cels (Figures 1G,I).

**FIGURE 2:**
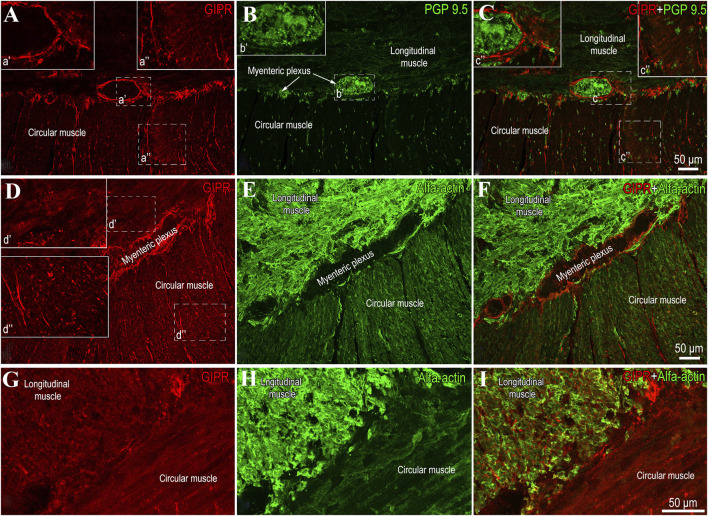
Representative images showing GIP receptor (GIPR) localization in the human jejunal muscular tissue. **(A–C)** demonstrate the absence of the GIPR (a’) in the myenteric plexus, which is immunolabeled with the general neuronal marker PGP 9.5 (b’, c’’). However, light immunoreactivity is visible in the circular muscle layer (a’’, c’’). **(D–F)** show a cross-section of the circular muscle layer and a longitudinal section of the longitudinal muscle layer. **(G–I)** provide a higher-magnification view of the longitudinal section of the circular muscle layer and the cross-section of the longitudinal muscles, highlighting the uniform distribution of GIPR in the smooth muscles.

In contrast to GLP-1 receptors, GIP receptors (GIPR) were not detected in either the submucosal or the myenteric plexus (Figures 2A–F). Immunohistochemical analysis showed that GIPR are located in the smooth muscle myocytes of the circular and longitudinal muscle layers (Figures 2D–I).

**FIGURE 3:**
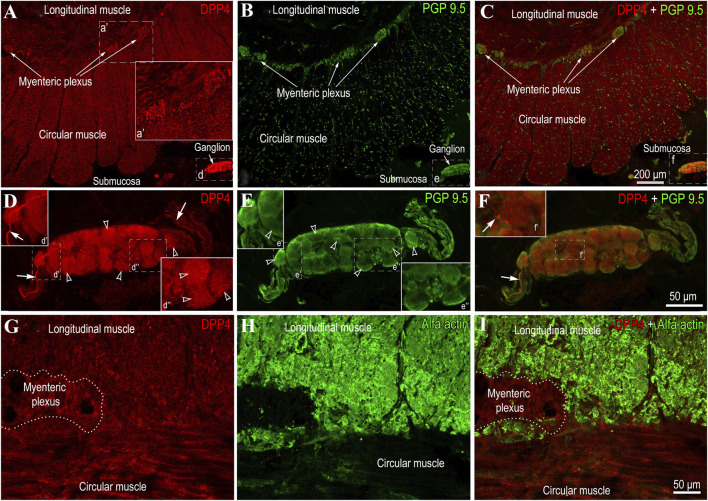
Representative images showing DPP-4 localization in the human jejunal muscular tissue **(A–C)** show a low-magnification view of the circular and longitudinal muscle layers, as well as the myenteric plexus between them. Notably, the concentration of DPP4 is slightly higher in the myenteric plexus, immunolabeled with the general neuronal marker PGP 9.5 **(B,C)**, compared to the muscular tissue. **(D–F)** depict the submucosal ganglion. The distribution of DPP4 in neurons (empty arrowheads) appears even though some fibers (**D**, d’, f’) are more intensely positive compared to others (white arrows). Boxed areas in the panels are enlarged in the corners. **(G–I)** provide a higher-magnification view of the circular and longitudinal muscle layers (stained with an anti-smooth muscle antibody) and the myenteric plexus (outlined by a dashed area) between them, highlighting the DPP4 signal in the tissues.

Immunofluorescence analysis demonstrated that DPP4 is localized in both neural and muscular tissues (Figure 3). The myenteric and submucosal ganglia showed immunopositivity for DPP4. However, in contrast to GLP-1R receptors, the entire neuronal body was displayed immunoreactivity (Figures 3D–F), while some nerve fibers connected to the ganglia or lying between neurons (Figures 3D,D’,f’) exhibited stronger positivity compared to others. Additionally, DPP4 positivity was detected in the smooth muscle myocytes of the circular and longitudinal muscle layers (Figures 3A–C,G–I).

### DPP-4 activity and protein expression of DPP-4, GLP-1, GIP, and their receptors in jejunal muscular tissue

**FIGURE 4:**
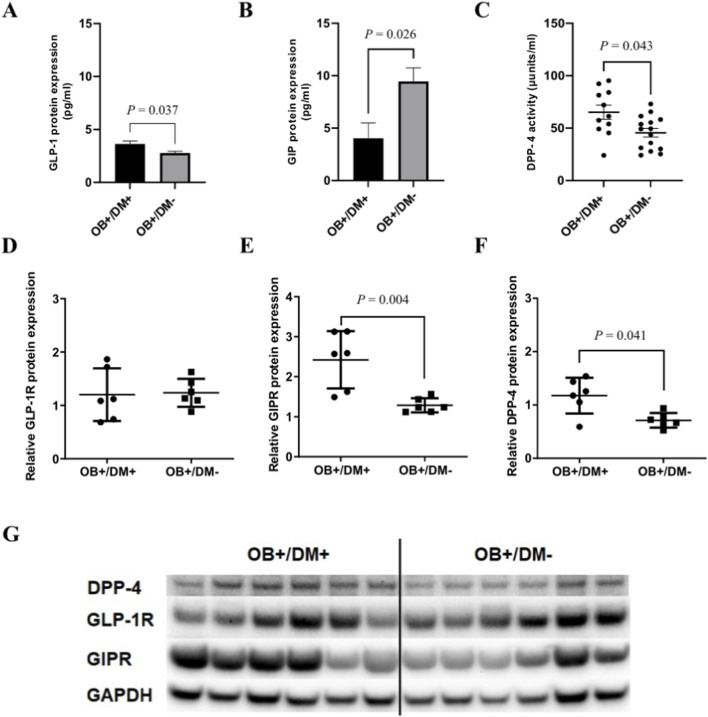
Protein expression of GLP-1, GIP, GLP-1R, GIPR, DPP-4, and DPP-4 activity in jejunal muscular tissue. Protein expression of **(A)** GLP-1 (OB+/DM+ n = 12; OB+/DM− n = 12), **(B)** GIP (OB+/DM+ n = 4; OB+/DM− n = 11), **(C)** DPP-4 enzymatic activity (OB+/DM+ n = 11; OB+/DM− n = 15), **(D)** GLP-1 receptor (GLP-1R), **(E)** GIP receptor (GIPR), and **(F)** DPP-4. Representative western blot images **(G)** showing expression of DPP-4, GLP-1R, GIPR, and GAPDH in OB+/DM+ (n = 6) and OB+/DM− (n = 6) tissues. Data presented as mean ± SEM. P ≤ 0.05 (Mann-Whitney U-test).

The relative GLP-1 protein expression in jejunal muscular tissue showed significant tendency to increase in obese patients with diabetes (OB+/DM+: 3.62 ± 0.98 pg/mL) compared to obese patients without diabetes (OB+/DM−: 2.77 ± 0.54 pg/mL), as shown in Figure 4A (*P* = 0.037).

In contrast, GIP protein expression was significantly higher in the OB+/DM− group (9.46 ± 4.27 pg/mL) than in OB+/DM+: patients (4.03 ± 2.91 pg/mL; *P* = 0.026) (Figure 4B), indicating a potential diabetes-associated reduction in jejunal GIP content.

Furthermore, DPP-4 enzymatic activity in jejunal muscular tissue was also significantly elevated in OB+/DM+ patients (65.12 ± 22.01 U/mL) compared to OB+/DM− patients (45.57 ± 15.80 U/mL; *P* = 0.042) (Figure 4C), suggesting altered peptide metabolism in diabetic condition.

The results of GLP-1 receptor (GLP-1R), GIP receptor (GIPR) and DPP-4 relative expression in jejunal muscular tissue show the same levels of GLP-1R in both OB+/DM+ and OB+/DM- groups (*P* > 0,05) (Figure 4D). As for GIPR and DPP-4, OB+/DM+ group had a significantly higher expression of both GIPR (OB+/DM+: 2.42 ± 0.71; OB+/DM−: 1.29 ± 0.18) (*P* = 0.004) and DPP-4 (OB+/DM+: 1.18 ± 0.34; OB+/DM−: 0.71 ± 0.14) (*P* = 0.041) (Figures 4E,F).

### Assessment of jejunal muscle contraction force

**FIGURE 5:**
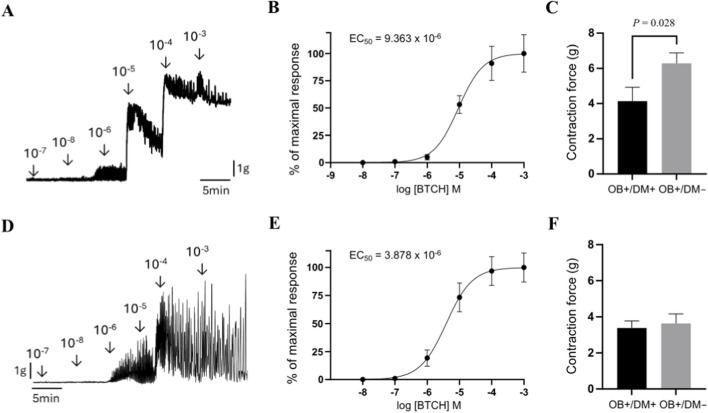
Assessment of BTCH-induced contraction force in human jejunal muscle strips. Representative original tracing of cumulative BTCH application (10^−8^ to 10^−3^ M) to jejunal muscle strips from non-diabetic patients (longitudinal muscle, top row; circular muscle, bottom row) **(A,D)**. Concentration–response curves (n = 7 per group) showing BTCH-induced contraction in longitudinal **(B)** and circular **(E)** muscle, responses are expressed as a percentage of the maximal contraction induced by 10^−3^ M BTCH. Comparison of BTCH-induced contraction force at a near-maximal concentration (10^−4^ M) in longitudinal muscle from patients with obesity and diabetes (OB+/DM+, n = 10) and from obese non-diabetic patients (OB+/DM−, n = 11) **(C)**. Comparison of BTCH-induced contraction force at a near-maximal concentration (10^−4^ M) in circular muscle from patients with obesity and diabetes (OB+/DM+, n = 12) and from obese non-diabetic patients (OB+/DM−, n = 12) **(F)**. Contraction force is expressed in grams **(G)**. Data are mean ± SEM. Statistical significance: P ≤ 0.05 (Mann–Whitney U-test).

The cumulative application of BTCH (10^−8^ to 10^−3^ M) to jejunal longitudinal muscle strips from non-diabetic patients induced a concentration-dependent increase in contraction force (Figure 5A). The corresponding concentration–response curve, generated using samples from 7 patients, demonstrated that BTCH evokes a sigmoidal contractile response, with an estimated EC_50_ value of 9.36 × 10^−6^ M (Figure 5B). Based on this curve, 10^−4^ M was selected as a near-maximal effective concentration for subsequent comparative experiments between patient groups.

When stimulated with 10^−4^ M BTCH, longitudinal muscle strips from patients with obesity but without diabetes (OB+/DM−; n = 11) exhibited a significantly higher contraction force (6.29 ± 1.96 g) compared to strips from patients with both obesity and diabetes (OB+/DM+; n = 10, 4.14 ± 2.52 g; *P* = 0.02) (Figure 5C).

Similar to the longitudinal muscle layer, cumulative application of BTCH (10^−8^ to 10^−3^ M) to jejunal circular muscle strips from non-diabetic patients induced a concentration-dependent increase in contraction force (Figure 5A). The EC_50_, calculated from the concentration–response curve generated using samples from n = 7 patients, was 3.88 × 10^−6^ M (Figure 5B). Responses were normalized to the maximal contraction induced by 10^−3^ M BTCH, and 10^−4^ M was used as a near-maximal concentration for group comparison.

At this concentration, circular muscle strips from OB+/DM+ (n = 12) and OB+/DM− (n = 12) patients generated comparable contraction forces (3.39 ± 1.35 g and 3.64 ± 1.81 g, respectively), with no statistically significant difference between groups (*P* > 0.05) (Figure 5F).

### Effects of GLP-1 and GIP on BTCH-induced contractions in jejunal muscle layers

**FIGURE 6:**
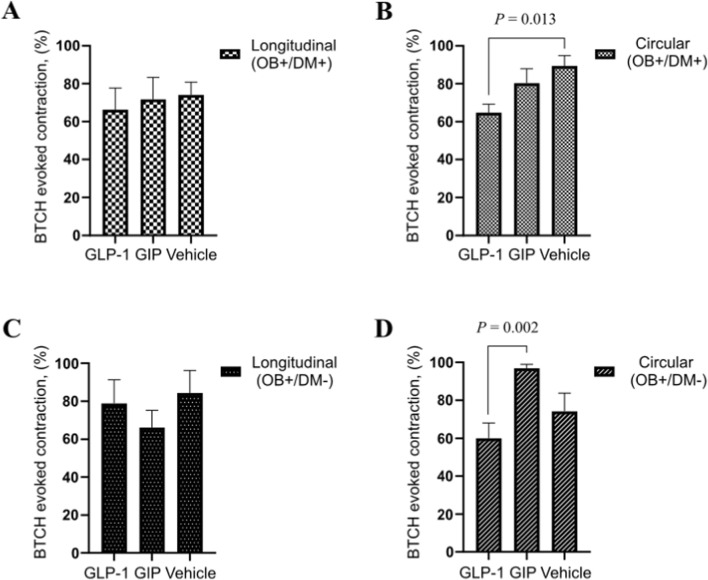
Experimental design and representative data presentation for GLP-1 and GIP effects on BTCH-induced jejunal muscle contraction. BTCH (10^−4^ M)-evoked contractions in longitudinal **(A)** and circular **(B)** jejunal muscle strips from (OB+/DM+) patients after incubation with GLP-1 (10^−7^ M; n = 7 for longitudinal, n = 6 for circular), GIP (10^−7^ M; n = 8 for both), or vehicle (n = 8 for both). **(C,D)** present corresponding experimental conditions in longitudinal **(C)** and circular **(D)** muscle strips from (OB+/DM–) patients incubated with GLP-1 (10^−7^ M; n = 6), GIP (10^−7^ M; n = 6), or vehicle (n = 6). Contractile responses expressed as percentages of BTCH-induced maximal contraction were compared between GLP-1, GIP and vehicle, and only significant differences by *P* ≤ 0.05 (Mann-Whitney U-test) were denoted in the Figures.

In patients with obesity and diabetes (OB+/DM+), neither GLP-1 (66.31% ± 30.11%, n = 7) nor GIP (71.74% ± 32.57%, n = 8) significantly altered BTCH-induced contraction in longitudinal muscle strips compared to control (vehicle-treated, 74.08% ± 18.93%, n = 8; *P* > 0.05) (Figure 6A). In contrast, in circular muscle strips from the same group, GLP-1 significantly inhibited the BTCH-evoked contraction compared to control (64.82% ± 10.83% vs. 89.39% ± 15.37%, n = 6 and 8 respectively; *P* = 0.013) (Figure 6B).

In patients with obesity but without diabetes (OB+/DM−), no significant changes in longitudinal muscle contraction were observed following incubation with either GLP-1 (78.87% ± 30.47%, n = 6) or GIP (66.20% ± 22.02%, n = 6) when compared to control (84.38% ± 28.92%, n = 6; *P* > 0.05) (Figure 6C). However, in circular muscle strips from OB+/DM− patients, GLP-1 significantly inhibited BTCH-induced contraction compared to GIP (60.07% ± 19.73% vs. 96.90% ± 5.02%, n = 6; *P* = 0.002), suggesting a stronger inhibitory effect of GLP-1 in this muscle layer (Figure 6D). TTX application had no measurable effect on the modulatory actions of GLP-1 or GIP on jejunal contractility suggesting that the effect of the peptides is direct on the muscle cells and not via nerves. As the responses remained consistent in both TTX-treated and untreated strips, the data is not presented.

## Discussion

In the present study GLP-1 receptors (GLP-1R) were found not only in the nuclei of submucosal and myenteric plexuses neurons, but also in the nuclei of smooth muscle cells in both circular and longitudinal layers of jejunal muscular tissue. In contrast, GIP receptors (GIPR) were localized only in the circular and longitudinal muscular layers of jejunum. DPP-4 was present throughout neural and muscular tissues of jejunum. Expression of GIPR was significantly higher and expression of GIP protein significantly lower in the jejunal muscular tissue of the patients with T2DM. In comparison, GLP-1 protein expression and DPP-4 activity was significantly higher among patients with T2DM. The contraction force evoked by BTCH was significantly weaker in the longitudinal muscle of patients with T2DM and GLP-1 significantly reduced contractions of circular, but not longitudinal muscles in patients with and without T2DM.

The incretin hormones GLP-1 and GIP are secreted by L and K enteroendocrine cells, particularly following meal ingestion (Gutierrez-Aguilar and Woods, 2011). Within the local gut environment, the concentrations of these incretins can be notably elevated, potentially surpassing their levels in circulation. This localized, direct paracrine activity has generated considerable interest in understanding how physiological and pharmacological concentrations of GLP-1 and GIP may influence key tissues implicated in the pathophysiology of conditions such as diabetes. Although these hormones are traditionally considered to exert their effects on peripheral organs via the bloodstream, GLP-1 and GIP are rapidly degraded upon entering circulation by the soluble form of DPP4 (Morrow et al., 2021). Additionally, these hormones are inactivated locally in the gut by the membrane-bound form of DPP4 (Kamakura et al., 2020). However, the relative contributions of these two forms of DPP4 to incretin inactivation remain poorly understood.

DPP-4 has been extensively reported to be expressed in the muscular layer of the jejunum and is associated with local levels of NPY family peptides, including neuropeptide Y (NPY), peptide YY (PYY), and pancreatic polypeptide (PP) (Malinauskas et al., 2025). In the present study, we demonstrate that DPP4 is present in both neural and muscular longitudinal and circular layers. The interplay between the local concentration of GLP-1 and GIP and its receptors, in relation to DPP4 expression, is therefore critical for maintaining physiological activity within the intestine. Moreover, evidence suggests that GLP-1, cleaved by DPP4, might act as a weak partial agonist or antagonist of GLP-1R, potentially eliciting physiological effects such as vasodilation (McLean et al., 2021).

Targeting GLP-1R through GLP-1 actions is challenging, as many tissues and cellular targets influenced by GLP-1 lack robust or detectable GLP-1R expression. GLP-1R has been identified in vagal afferent neurons (McLean et al., 2021). GLP-1 is expressed in immune cells, the intestinal vasculature, and enteric neurons within the intestine, where it may mediate immediate paracrine effects (McLean et al., 2021; Richards et al., 2014). In this study, we demonstrate that GLP-1R is strongly expressed in the nuclei of smooth muscle cells as well as within the myenteric plexus and submucosal ganglia. In contrast, GIP receptors were not observed in the submucosal ganglia or the myenteric plexus but were identified in smooth muscle myocytes within both the circular and longitudinal muscle layers. The localization of GIP-R in the gastrointestinal tract remains largely unresolved, though previous studies have identified its presence on the basolateral surface of epithelial cells in the proximal jejunum (Morrow et al., 2021).

The expression of GLP-1R in enteric nerves and intestinal muscular tissue layers is particularly significant when evaluating the physiological effects of food intake and bariatric surgery. Gastric bypass surgery leads to a substantial elevation in GLP-1 levels, and antagonism of GLP-1 largely negates the postsurgical improvements observed in glucose-stimulated insulin secretion (Jirapinyo et al., 2018). The physiological implications of endogenous GLP-1, which is released locally at high concentrations following surgery and may reach elevated levels at the sites of nerve endings or smooth muscle cells, warrant further investigation.

Previous studies have demonstrated that GLP-1 and GIP exert pleiotropic effects not only systemically but also locally in the gastrointestinal tract, including potential direct actions on smooth muscle and the enteric nervous system (Brubaker and Drucker, 2002). However, detailed analysis of their expression in the muscular layer of the human jejunum remains limited. In this study, we observed that GLP-1R protein expression in the jejunal muscular tissue did not significantly differ between obese individuals with and without T2DM. This finding suggests that GLP-1R abundance may remain stable regardless of diabetic status, in line with earlier findings showing widespread expression of GLP-1R in the gastrointestinal tract (Pyke et al., 2014). However, some animal studies have reported a downregulation of GLP-1 receptors in response to high glucose and GLP-1 levels in T2DM models, which may reflect interspecies differences or context-dependent regulatory mechanisms that are not necessarily applicable to humans (Xu et al., 2007; Rajan et al., 2015). In contrast, GIP receptor (GIPR) expression was significantly reduced in OB+/DM− compared to OB+/DM+ individuals. This may indicate diabetes-associated upregulation of GIPR in the jejunal muscle in response to reduced tissue GIP levels, as demonstrated in the present study.

GLP-1 protein levels in the jejunal muscular layer were increased in the OB+/DM+ group compared with OB+/DM− individuals, whereas GIP levels were decreased. It is known that the degradation of both peptides depends on DPP-4 activity. In our study, DPP-4 protein expression and enzymatic activity were significantly higher in OB+/DM+ compared with OB+/DM− subjects, consistent with previous reports describing increased DPP-4 expression and activity in obesity and T2DM (Barchetta et al., 2019; Sell et al., 2013). The reduction in GIP levels is consistent with enhanced DPP-4 activity, while the increase in GLP-1 cannot be explained by this mechanism based on the current data.

In the gastrointestinal system smooth muscle cells are exposed to excitatory and inhibitory effects of different peptides and neurotransmitters. Acetylcholine acts through five muscarinic receptors, however, on smooth muscle cell surface mainly M2 and M3 subtypes are expressed in the proportion of 3–5 to 1 (Caulfield, 1993). M3 receptors are coupled to the G protein Gq/G11and through phospholipase C pathway mediates smooth muscle contraction. Contrary to M3, M2 receptors are coupled to the G protein Gi/Go, which inhibits adenyl cyclase and causes relaxation of the smooth muscle (Tanahashi et al., 2021). Similarly, GLP-1R in myenteric neurons is coupled to the G-protein Gsα and through adenylyl cyclase inhibits gastrointestinal motility (Halim et al., 2018). The results of the current study show that GLP-1 significantly reduced BTCH evoked contractions of the circular, but not longitudinal jejunal muscles in patients with or without T2DM. This effect was attributable to a direct action on the muscle cells, as blockade of neural transmission with TTX did not produced a significant influence. Previous human (Tanahashi et al., 2021) and experimental animal studies (Amato et al., 2010; Amato et al., 2014) have demonstrated similar results in the smooth muscles of duodenum and colon. Circular muscle contractions are dominant in peristalsis and excessive contractions may even cause abdominal cramps. Inhibitory effect of GLP-1 may contribute to increased intestinal transit time and constipation, the main symptoms also present in the patients having glucagonlike peptide secretory tumor (Chen et al., 2013). Furthermore, randomized controlled study investigating the effects of GLP-1 analogue ROSE-010 in the treatment of patients with IBS found, that ROSE-010 have caused an effective relief of acute pain attacks (Brubaker et al., 2002). Thus, the GLP-1 and its analogues could be efficient in the treatment of abdominal cramps by inhibiting excessive contractions of the circular muscle of the intestine even with near maximal acetylcholine concentrations. It is also worth mentioning that GIP had no effect on contraction force of jejunal muscle, confirming previous findings from the rats, that GLP-1 was about 2.6–16.7 times more potent than GIP in abolishing the migrating myoelectric complex of the small intestine (Edholm et al., 2009).

Gastrointestinal dysfunction is a common finding among patients with T2DM. Recent multi-segmental magnetic resonance imaging study comparing patients with T2DM and healthy controls have found that patient T2DM group had 40% larger small bowel volume, less small bowel motility postprandially and there were positive associations between small bowel motility and constipation scores (Hellström et al., 2009). Diabetic neuropathy may affect myenteric neurons and result in abnormal intestinal motility. The studies investigating motility in the jejunum and ileum of the rats or guinea-pigs found hypercontractility of longitudinal smooth muscles to stimulation with acetylcholine agonist carbachol (Bertoli et al., 2023; Carrier and Aronstam, 1990; Narimatsu et al., 2007; Cellini et al., 2012). Such an effect may partially be explained by the upregulation of M2 and M3 receptors in diabetic rat ileum (Carrier and Aronstam, 1990). In contrast, the results of our study show that in humans acetylcholine analog (BTCH) induced contractions were significantly weaker in jejunal longitudinal smooth muscle of patients with T2DM. The pathology study in patients with diabetic gastroparesis found that most common histologic abnormalities of the gastric muscle were loss of interstitial cells of Cajal (ICC) with remaining ICC showing injury (Kim et al., 2011). Furthermore, in diabetic rat study decreased number of ICC networks resulted in significantly weaker contractility of the proximal colon (Grover et al., 2011). In current study we did not investigate histological changes in jejunal smooth muscles, thus future clinical studies in diabetic patients are needed to give more insight into the possible mechanisms of gastrointestinal dysfunction. Furthermore, although TTX had no effect on our results, it is still not fully understood whether the activation of GLP-1R or GIP-R inhibits gut motility indirectly through neural pathways or via direct interactions with smooth muscle. GLP-1R remains unclear and warrants further investigation.

## Conclusion

This study identifies distinct expression patterns and functional effects of GLP-1 and GIP in jejunal muscle from obese individuals with and without T2DM. GLP-1R was detected in both smooth muscle and enteric plexuses, but the TTX-insensitive inhibition of contraction indicates that GLP-1 acts predominantly through a myogenic mechanism. In contrast, GIPR was confined to smooth muscle and showed no functional effect.

Overall, reduced cholinergic activity in longitudinal muscle, lower GIP, and increased GLP-1 in T2DM indicate a shifted local incretin environment that may collectively suppress jejunal contractility.

## Data Availability

The original contributions presented in the study are included in the article/supplementary material, further inquiries can be directed to the corresponding author.

## Abbreviations

AMC, 7-amino-4-methylcoumarin; BMI, body mass index; BTCH, bethanechol chloride; DMSO, dimethyl sulfoxide; DPP-4, dipeptidyl peptidase-4; ELISA, enzyme-linked immunosorbent assay; GAPDH, glyceraldehyde-3-phosphate dehydrogenase; GIP, glucose-dependent insulinotropic polypeptide; GIPR, glucose-dependent insulinotropic polypeptide receptor; GLP-1, glucagon-like peptide-1; GLP-1R, glucagon-like peptide-1 receptor; HbA1c, glycated hemoglobin A1c; HDL, high-density lipoprotein; IBS, irritable bowel syndrome; M1–M5, muscarinic acetylcholine receptor subtypes 1–5; M2, muscarinic acetylcholine receptor subtype 2; M3, muscarinic acetylcholine receptor subtype 3; NO, nitric oxide; OB+/DM+, obese patients with type 2 diabetes mellitus; OB+/DM−, obese patients without type 2 diabetes mellitus; PBS, phosphate-buffered saline; PFA, paraformaldehyde; PVDF, polyvinylidene difluoride; SDS–PAGE, sodium dodecyl sulfate–polyacrylamide gel electrophoresis; SGLT2, sodium–glucose cotransporter 2; SPSS, Statistical Package for the Social Sciences; T2DM, type 2 diabetes mellitus; TTX, tetrodotoxin
